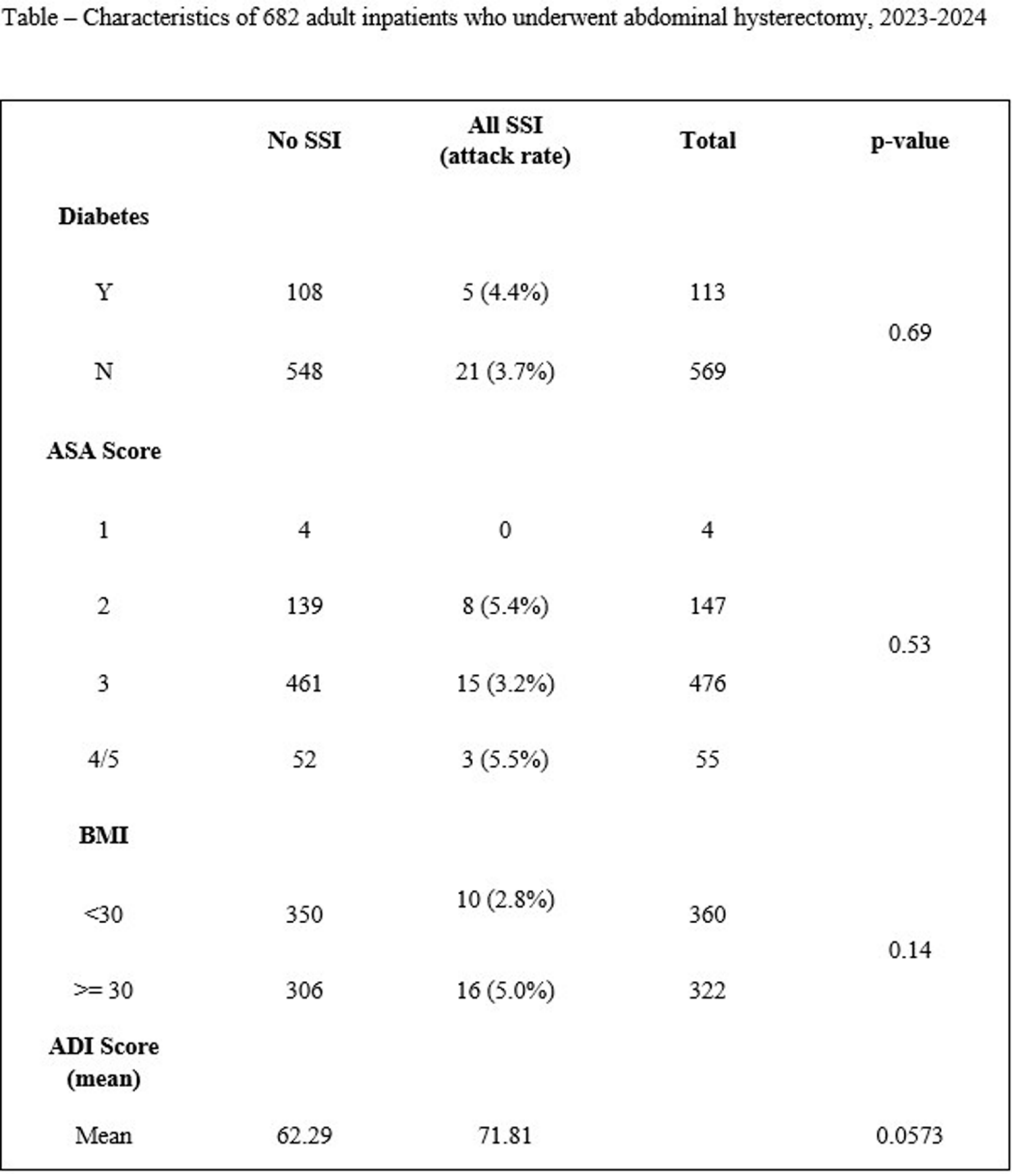# 102 Barriers and Facilitators to Infection Prevention in Behavioral Health Settings

**DOI:** 10.1017/ash.2026.10719

**Published:** 2026-06-23

**Authors:** Melanie Zarnoski, Patrick Burke, Thomas Fraser, Petros Svoronos

**Affiliations:** 1 Cleveland Clinic; 2 Cleveland Clinic Foundation

## Abstract

**Background:** Socio-economic factors may play a role in patients’ risk for healthcare associated infections (HAI), including surgical site infections (SSI). The Area Deprivation Index (ADI) is a mapping tool that characterizes neighborhood-level socioeconomic disadvantage based on income, education, employment, and housing quality. A higher ADI score indicates a greater disadvantage. ADI has been studied extensively as a predictor for various patient outcomes. The purpose of this study was to establish whether an association exists between the national ADI score and SSI following abdominal hysterectomy at a 1400-bed academic center in northeast Ohio. **Methods:** At the Cleveland Clinic, SSIs are identified by Infection Preventionists using NHSN surveillance definitions. Using our surveillance software (EPIC, Bugsy, Verona, Wisconsin), we extracted all NHSN-defined inpatient abdominal hysterectomy procedures in adult patients between January 1, 2023 and December 31, 2024, including those with and without an SSI. We queried our electronic medical record data warehouse for the national ADI score recorded closest to the surgical procedure. Patients without an ADI score were excluded. Characteristics of patients with and without a SSI were summarized. Categorical variables were compared using a chi-square test and a two-tailed t-test was used to compare mean ADI scores. **Results:** During the study period 682 patients met criteria that underwent an inpatient abdominal hysterectomy, 26 developed an NHSN-defined SSI, including 15 superficial incisional SSI and 11 organ space SSI. Counts and attack rates of SSI, stratified by relevant factors are shown in the Table. Patients with an SSI had an average ADI of 71.81 while patients with no SSI had an average ADI of 62.29 (p=0.0573). **Conclusion:** In this study, the mean ADI trended higher for patients who developed a SSI following an abdominal hysterectomy, indicating a possible association between socio-economic factors and risk of a SSI. Although the test for difference in mean ADI did not achieve statistical significance, it highlights ADI as a readily obtainable patient characteristic that may contribute to post-operative infection risk. Further exploration of obtainable patient characteristics, including ADI score, is beneficial to inform best practice for optimal outcomes following an abdominal hysterectomy procedure.

**Figure:**